# Agreement Between Prospective and Retrospective Measures of Childhood Maltreatment: A Systematic Review and Meta-analysis

**DOI:** 10.1001/jamapsychiatry.2019.0097

**Published:** 2019-03-20

**Authors:** Jessie R. Baldwin, Aaron Reuben, Joanne B. Newbury, Andrea Danese

**Affiliations:** 1 Social, Genetic and Developmental Psychiatry Centre, King’s College London, London, United Kingdom; 2 Department of Psychology and Neuroscience, Duke University, Durham, North Carolina; 3 Department of Child and Adolescent Psychiatry, Institute of Psychiatry, Psychology and Neuroscience, King’s College London, London, United Kingdom; 4 National and Specialist CAMHS (Child and Adolescent Mental Health Services) Trauma, Anxiety, and Depression Clinic, South London and Maudsley NHS (National Health Service) Foundation Trust, London, United Kingdom

## Abstract

**Importance:**

Childhood maltreatment is associated with mental illness. Researchers, clinicians, and public health professionals use prospective or retrospective measures interchangeably to assess childhood maltreatment, assuming that the 2 measures identify the same individuals. However, this assumption has not been comprehensively tested.

**Objective:**

To meta-analyze the agreement between prospective and retrospective measures of childhood maltreatment.

**Data Sources:**

MEDLINE, PsycINFO, Embase, and Sociological Abstracts were searched for peer-reviewed, English-language articles from inception through January 1, 2018. Search terms included child* maltreatment, child* abuse, child* neglect, child bull*, child* trauma, child* advers*, and *early life stress* combined with prospective* and *cohort*.

**Study Selection:**

Studies with prospective measures of childhood maltreatment were first selected. Among the selected studies, those with corresponding retrospective measures of maltreatment were identified. Of 450 studies with prospective measures of childhood maltreatment, 16 had paired retrospective data to compute the Cohen κ coefficient.

**Data Extraction and Synthesis:**

Multiple investigators independently extracted data according to PRISMA and MOOSE guidelines. Random-effects meta-analyses were used to pool the results and test predictors of heterogeneity.

**Main Outcomes and Measures:**

The primary outcome was the agreement between prospective and retrospective measures of childhood maltreatment, expressed as a κ coefficient. Moderators of agreement were selected a priori and included the measure used for prospective or retrospective assessment of childhood maltreatment, age at retrospective report, sample size, sex distribution, and study quality.

**Results:**

Sixteen unique studies including 25 471 unique participants (52.4% female [SD, 10.6%]; mean [SD] age, 30.6 [11.6] years) contained data on the agreement between prospective and retrospective measures of childhood maltreatment. The agreement between prospective and retrospective measures of childhood maltreatment was poor, with κ = 0.19 (95% CI, 0.14-0.24;* P* < .001). Agreement was higher when retrospective measures of childhood maltreatment were based on interviews rather than questionnaires (Q = 4.1521; *df* = 1; *P* = .04) and in studies with smaller samples (Q = 4.2251; *df* = 1; *P* = .04). Agreement was not affected by the type of prospective measure used, age at retrospective report, sex distribution of the sample, or study quality.

**Conclusions and Relevance:**

Prospective and retrospective measures of childhood maltreatment identify different groups of individuals. Therefore, children identified prospectively as having experienced maltreatment may have different risk pathways to mental illness than adults retrospectively reporting childhood maltreatment. Researchers, clinicians, and public health care professionals should recognize these critical measurement differences when conducting research into childhood maltreatment and developing interventions.

## Key Points

**Question:**

What is the agreement between prospective and retrospective measures of childhood maltreatment?

**Findings:**

This systematic review and meta-analysis of 16 unique studies and 25 471 unique participants found poor agreement between prospective and retrospective measures of childhood maltreatment, with Cohen κ = 0.19. On average, 52% of individuals with prospective observations of childhood maltreatment did not retrospectively report it, and likewise, 56% of individuals retrospectively reporting childhood maltreatment did not have concordant prospective observations.

**Meaning:**

Because findings from this meta-analysis demonstrated that prospective and retrospective measures of childhood maltreatment identify largely different groups of individuals, the 2 measures cannot be used interchangeably to study the associated health outcomes and risk mechanisms.

## Teaser

This systematic review and meta-analysis assesses the agreement between prospective and retrospective measures of childhood maltreatment.

## Introduction

Do prospective and retrospective measures of childhood maltreatment identify the same individuals? This question has captivated psychiatrists and psychologists since the inception of our discipline^1^ and still permeates many aspects of our professions. Researchers use retrospective reports as a shortcut to better understand the consequences of childhood maltreatment without the significant time or financial investment needed to undertake cohort studies.^2^ Clinicians use retrospective reports to swiftly identify individuals who are at heightened risk of mental illness by virtue of their exposure to childhood maltreatment.^3^ Public health professionals use retrospective reports to pragmatically estimate the health burden associated with exposure to childhood maltreatment.^4^ All these practices rely on the assumption that retrospective reports and prospective measures identify the same, or at least similar, groups of individuals. However, qualitative reviews^5,6^ have raised concerns about the validity of this assumption. Herein we present, to our knowledge, the first quantitative assessment of the agreement between retrospective reports and prospective measures of childhood maltreatment.

## Methods

### Data Sources

We performed a systematic review and meta-analysis in line with the PRISMA and MOOSE guidelines, following an a priori–defined protocol (eMethods and eTables 1 and 2 in the Supplement). We searched MEDLINE, PsycINFO, Embase, and Sociological Abstracts for peer-reviewed articles written in English and published from database inception to January 1, 2018, that included prospective assessments of childhood maltreatment. We used the following search terms: child* maltreatment, child* abuse, child* neglect, child bull*, child* trauma, child* advers*, and *early life stress* combined with prospective* and *cohort*.

### Study Selection

Two authors (J.R.B. and A.R.) independently screened titles and abstracts of all articles retrieved from the search before reviewing the full text of potentially eligible studies. We included original, peer-reviewed articles with prospectively collected information on childhood maltreatment (age <18 years). Measures of maltreatment (sexual abuse, physical abuse, emotional abuse, and neglect), domestic violence, bullying, institutionalization, and broader measures of adverse childhood experiences that included maltreatment were used to define overall childhood maltreatment. From the articles with prospective assessment of childhood maltreatment, we selected studies with data on corresponding retrospective measures (defined as subsequent assessment of the same individuals’ exposure undertaken at any age).

### Data Extraction

Three authors (J.R.B., A.R., and J.B.N.) independently extracted data from all studies with prospective assessment of childhood maltreatment on sample characteristics (cohort name, sample size, location, age at latest assessment, and sex distribution), childhood maltreatment type(s) assessed, prospective measure type(s) (official records, interview, and questionnaire), source (child protection services, hospital records, parent, child, teacher, or multiple informants), and availability of retrospective measures. If retrospective measures of childhood maltreatment were available, 2 authors (J.R.B. and A.D.) subsequently extracted data on the retrospective measurement type (interview or questionnaire) and source, agreement between prospective and retrospective measures, and study quality. Inconsistencies were resolved in consensus meetings and confirmed with the authors of the primary studies when necessary. Relevant missing information was requested from authors.

### Statistical Analysis

The extracted data were converted to contingency tables comparing prospectively identified childhood maltreatment (yes or no) with retrospectively reported childhood maltreatment (yes or no). From the contingency tables, we derived estimates of prevalence, raw percentage of agreement between measures, and Cohen κ coefficient. Some studies only reported a κ. Prevalence and raw percentage of agreement estimates were used for descriptive purposes, and our primary outcome was the κ. The other extracted variables were used to explain the heterogeneity in the κs across studies.

We first described the prevalence of childhood maltreatment based on prospective and retrospective measures of childhood maltreatment. We then examined the (1) prevalence of retrospective reports of childhood maltreatment among those with prospective observations, (2) prevalence of prospective observations among those with retrospective reports, and (3) raw percentage of agreement between the 2 measures through meta-analyses of proportions for different childhood maltreatment types with the metafor R package.^7^ Data from contingency tables were first converted using the Freeman-Tukey double arcsine transformation^8^ to normalize and stabilize the variance of the sampling distribution, then aggregated using random-effects model meta-analyses, and finally back-transformed using the inverse of the Freeman-Tukey double arcsine transformation.^8^ To display the overlap between prospective and retrospective measures of child maltreatment based on these meta-analyses, we built Venn diagrams using the VennDiagram R package.^9^ To build Venn diagrams, we let the relative complements (the prevalence of retrospective reports without prospective observations [R − P] and the prevalence of prospective observations without retrospective reports [P − R]) vary while holding the intersection (RΩP or the prevalence of concordant retrospective reports and prospective observations) constant.

Because the raw percentage of agreement can be inflated by chance, we derived a measure of agreement based on the κ, which accounts for chance findings and provides an estimate of variation in agreement in the population.^10^ The κs for each study were derived from contingency tables using the cohen.kappa() command from the psych R package,^11^ which computes CIs based on the variance estimates discussed by Fleiss et al.^12^ The meta-analyses of κs were undertaken with the metafor R package using a random-effects model. When a study reported multiple effect sizes for different types of maltreatment, we calculated the mean of multiple κs to generate a single overall effect size for each study. We also undertook a sensitivity analysis selecting the largest κ from each study to assess the upper limit of agreement.

We next explored the effects of various possible sources of artifact or bias on κ estimates using the metafor R package.^7^ First, we assessed heterogeneity between studies using the I^2^ statistic. Second, we assessed the presence of publication bias visually by funnel plot and formally by funnel plot–based tests, such as the Begg and Egger tests. Because these tests might be underpowered if only a few studies are available, we used a nonparametric trim-and-fill procedure to identify and correct for funnel plot asymmetry and reestimated the aggregate results. Third, we assessed the undue effect of individual studies on the meta-analysis results through jackknife sensitivity analyses, by testing changes in the estimate across permutations in which each study was omitted in turn.

Finally, we tested predictors of heterogeneity in κs. We used subgroup analyses to test the contribution of measurement characteristics (ie, measure used for prospective or retrospective assessment of maltreatment, type of childhood maltreatment). We also used metaregression analyses to test the contribution of sample characteristics (ie, sex distribution, age at retrospective report, sample size, and study quality) (eTable 4 in the Supplement for coding). A 2-tailed *P* < .05 was considered statistically significant.

## Results

### Search Results

**Figure 1.**
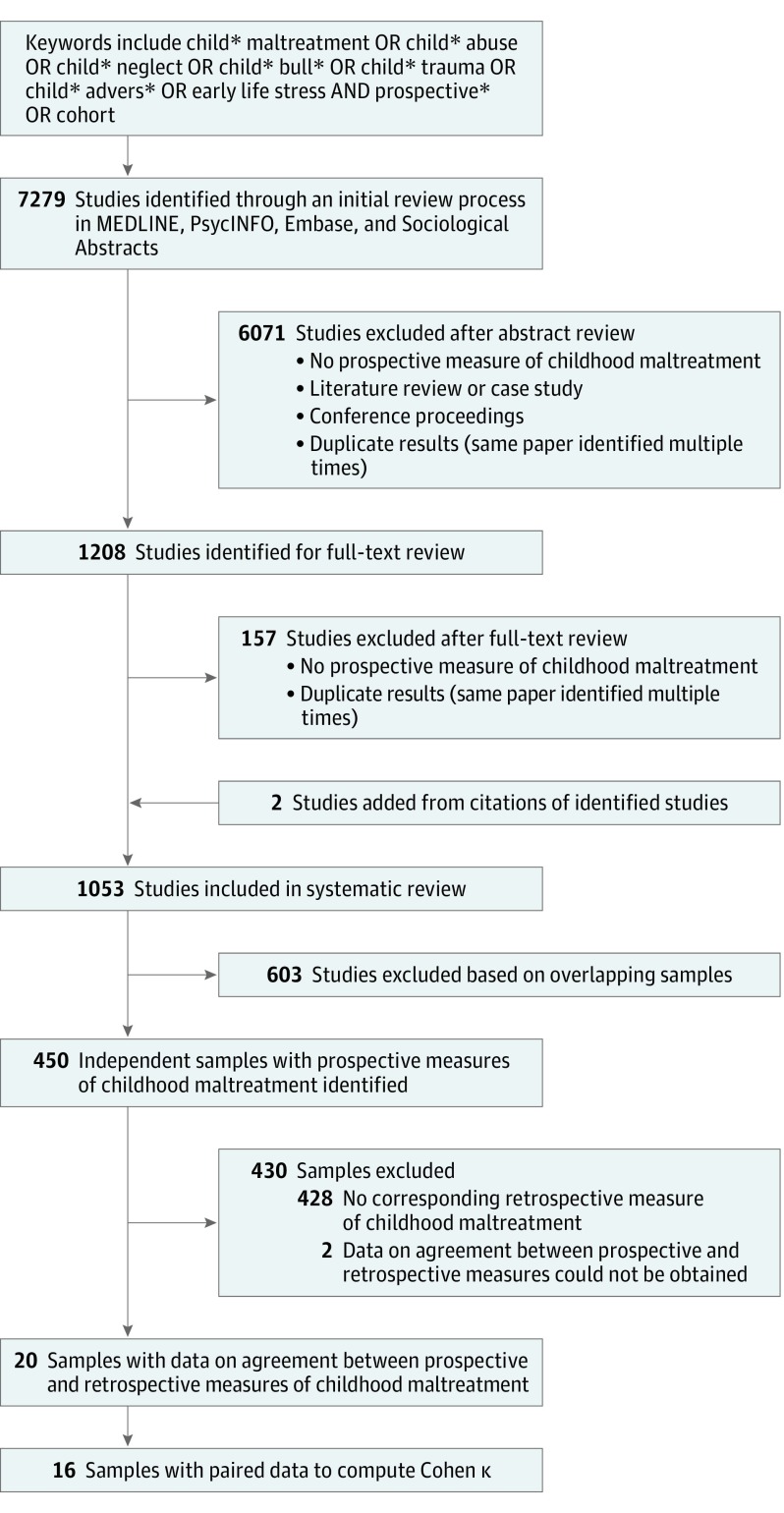
Study Selection for Meta-analysis of the Agreement Between Prospective and Retrospective Measures of Childhood Maltreatment A complete list of the studies included in the analysis with κ agreement is found in the Table.

The study selection procedure is summarized in Figure 1, and further details are provided in the eResults in the Supplement. We identified 450 independent studies with prospective measures of childhood maltreatment (eTable 5 in the Supplement). Of these studies, we identified 20 studies (26 365 participants) with at least partial data on the agreement between prospective and retrospective measures of childhood maltreatment and 16 unique studies^13,14,15,16,17,18,19,20,21,22,23,24,25,26,27,28^ with 25 471 unique participants (52.4% female [SD, 10.6%]; mean [SD] age, 30.6 [11.6] years) with direct measures or paired data sufficient to compute measures of κs. Details of these studies are reported in the Table.

**Table.** Description of Studies With Prospective and Retrospective Measures of Childhood Maltreatment

Source (Study Name)	No. of Participants	Female, %	Sample Description	Type of Maltreatment Assessed	Prospective Measure^a^		Retrospective Measure			Agreement Data	
					Type	Source	Type	Source	Age at Report, y	κ	Paired Measures
Robins,^29^ 1966 (Deviant Children Grown Up)	411	NA	Children with antisocial behavior problems and matched controls from St Louis, Missouri	Maltreatment	Mixed	CPS, self	Interview	Self	45	No	No
Williams,^30^ 1994	129	100	Sexually abused females from a Northeastern US city examined at a hospital, 1973-1975	Sexual abuse	Mixed	Medical records, self, parent	Interview	Self	24.5	No	No
Widom and Shepard,^31^ 1996; Widom and Morris,^13^ 1997; Raphael et al,^32^ 2001	1181-1196	48.7	Maltreated children and matched controls from a metropolitan area in the Midwestern United States	Maltreatment, physical abuse, and sexual abuse	Records	CPS	Interview	Self	28.7-29.2	Yes	Yes
Johnson et al,^14^ 1999 (Children in the Community Study)	639	47.7	Children from 2 counties in northern New York State	Maltreatment	Records	CPS	Interview	Self	22.3	Yes	Yes
Goodman et al,^33^ 2003	175	80.6	Sexually abused children referred from DAs’ offices in Denver, Colorado, 1985-1987	Sexual abuse	Records	CPS	Interview	Self	23	No	No
Tajima et al,^15^ 2004 (Lehigh Longitudinal Study)	409	45.7	Children from 2 counties in eastern Pennsylvania with overrepresentation from low-income families	Physical abuse	Interview	Parent	Interview	Self	18	Yes	Yes
White et al,^26^ 2007 (Rutgers Health and Human Development Project)	359	50.7	Adolescents from New Jersey recruited through random telephone sampling	Physical abuse	Questionnaire	Self	Questionnaire	Self	30.5	Yes	Yes
Everson et al,^16^ 2008 (Longitudinal Studies of Child Abuse and Neglect)	348-350	51	Children from Eastern and Southeastern United States identified to be high risk for poor medical or developmental outcomes	Physical, sexual, and emotional abuse	Records	CPS	Interview	Self	12	Yes	Yes
Shaffer et al,^17^ 2008 (Minnesota Longitudinal Study of Parents and Children)	125-170	47.1	Children born to pregnant women with low socioeconomic status who attended prenatal care in Minnesota	Maltreatment, physical and sexual abuse, and neglect	Mixed	CPS, self, parent, teacher	Interview	Self	19	Yes	Yes
Barnes et al,^34^ 2009	179	100	Sexually abused females and matched controls in Washington, DC	Sexual abuse	Records	CPS	Interview	Self	24	No	No
Scott et al,^18^ 2010 (The New Zealand Mental Health Survey)	2144	55.1	New Zealand, Maori, and Pacific adults in New Zealand	Maltreatment	Records	CPS	Interview	Self	21.9	Yes	Yes
Denholm et al,^19^ 2013 (National Child Development Study)^b^	8461	NA	Children from Great Britain born in a single week in 1958	Neglect	Multiple	Parent, teacher	Questionnaire	Self	45	Yes	Yes
Elwyn and Smith,^20^ 2013 (Rochester Youth Development Study)	846	27.1	Children from Rochester, New York, with overrepresentation of boys and students from high-crime census tracts	Maltreatment	Records	CPS	Interview	Self	22.7	Yes	Yes
Patten et al,^21^ 2015 (National Longitudinal Study Survey of Children and Youth/National Population Health Survey)	1977	48.3	Children from Canada assessed as part of 2 linked surveys in childhood and adulthood	ACEs	Interview	Parent	Interview	Self	NA	Yes	No
Plant et al,^27^ 2015 (South London Child Development Study)^b^	97	52.4	Children born to pregnant women who attended antenatal clinics in South London, UK	Physical and sexual abuse	Interview	Parent, self	Questionnaire	Self	25	Yes	Yes
Mills et al,^22^ 2016 (Mater-University of Queensland Study of Pregnancy)	3739	57.3	Children born to pregnant women who attended maternity services at a hospital in Brisbane, Australia	Sexual abuse	Records	CPS	Questionnaire	Self	21	Yes	Yes
Reuben et al,^23^ 2016 (Dunedin Multidisciplinary Health and Development Study)	950	49.9	Children born in Dunedin, New Zealand, in 1972-1973	Physical, sexual, and emotional abuse, neglect, and ACEs	Mixed	CPS, parent, research workers, teacher	Interview	Self	38	Yes	Yes
Shenk et al,^24^ 2016 (Female Adolescent Development Study)^b^	514	100	Maltreated females and matched controls from an area in the US Midwest	Maltreatment	Records	CPS	Interview	Self	15.7	Yes	Yes
Newbury et al,^25^ 2018 (E-Risk Longitudinal Twin Study)	2055	51	Twin children from England and Wales born in 1994-1995	Physical, sexual, and emotional abuse, neglect, and maltreatment	Interview	Parent, research worker	Interview	Self	18	Yes	Yes
Naicker et al,^28^ 2017 (Birth to Twenty Plus Cohort)	1506-1565	51.8	Children born in Soweto- Johannesburg, South Africa, during 7 weeks in 1990	Sexual, physical, and emotional abuse	Questionnaire	Self	Questionnaire	Self	23	Yes	Yes

Abbreviations: ACEs, adverse childhood experiences; CPS, child protection services; DA, district attorney; NA, not available.

^a^ “Mixed” refers to studies assessing childhood maltreatment using a combination of records and interviews or questionnaires; “multiple” refers to studies assessing childhood maltreatment using interviews and questionnaires (excluding records).

^b^ Data on agreement between prospective and retrospective measures were obtained from study authors rather than from the published article.

### Overlap Between Individuals Identified by Prospective or Retrospective Measures of Childhood Maltreatment

**Figure 2.**
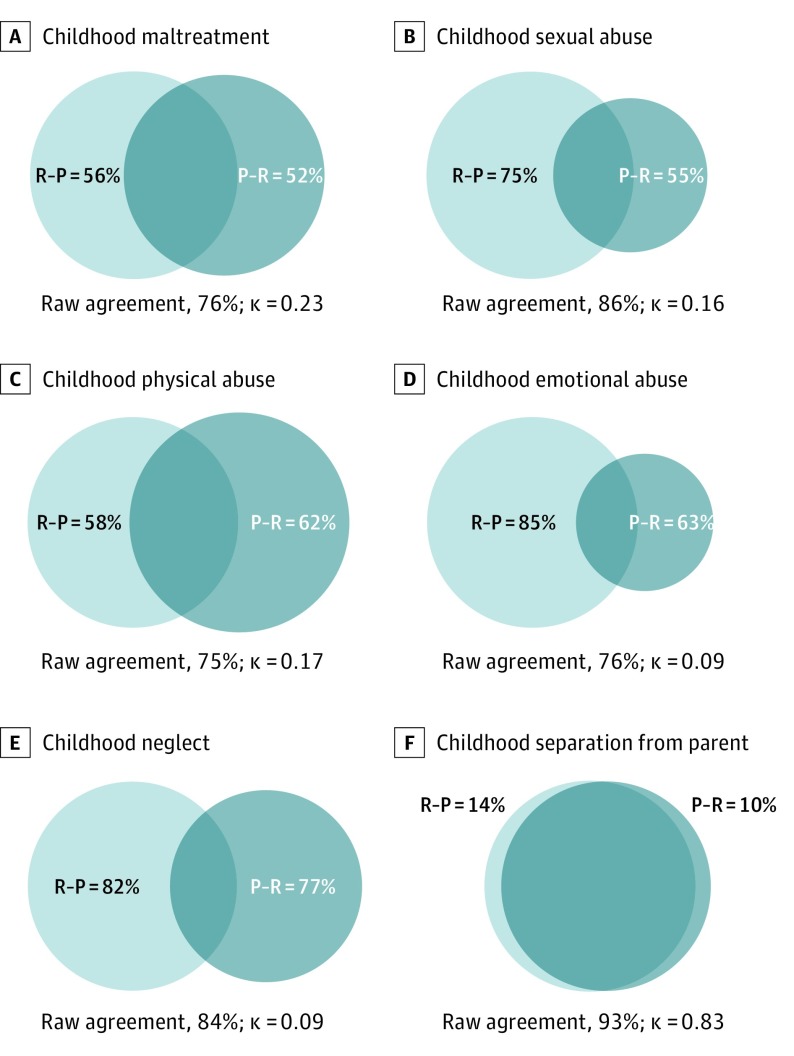
Overlap Between Individuals Identified by Virtue of Prospective or Retrospective Measures of Childhood Maltreatment In the Venn diagrams, the light circles indicate retrospective recall, whereas the dark circles indicate prospectively identified childhood maltreatment. The light nonoverlapping section (R-P) shows the proportion of individuals who retrospectively reported a history of childhood maltreatment but were not prospectively identified as experiencing maltreatment in childhood. The dark nonoverlapping section (P-R) shows the proportion of individuals who were prospectively identified as experiencing maltreatment in childhood but did not retrospectively report a history of childhood maltreatment. The overlap between the 2 circles (RΩP) shows the proportion of individuals who were prospectively identified as experiencing maltreatment in childhood and retrospectively reported a history of child maltreatment. Seven studies^14,17,18,20,24,25,32^ included childhood maltreatment; 8 studies,^13,16,17,22,23,25,27,28^ childhood sexual abuse; 9 studies,^15,16,17,23,25,26,27,28,31^ childhood physical abuse; 4 studies,^16,23,25,28^ childhood emotional abuse; and 4 studies,^17,19,23,25^ childhood neglect. An individual study by Reuben et al^23^ investigated the overlap between groups identified by virtue of prospective or retrospective measures of childhood separation from from parents (due to separation, divorce, death, or removal from home; not included in the meta-analysis).

eFigure 1 in the Supplement displays the range of prevalence estimates for childhood maltreatment based on 32 paired prospective and retrospective measures extracted from 15 studies.^14,15,16,17,18,19,20,22,23,24,25,26,27,28,31^ Capitalizing on the paired nature of the data, we next analyzed (1) the prevalence of retrospective reports of childhood maltreatment among those with prospective observations, (2) the prevalence of prospective observations among those with retrospective reports, and (3) the raw percentage of agreement between prospective and retrospective measures. A random-effects meta-analysis of 7 studies^14,17,18,20,24,25,32^ focusing on a broad measure of child maltreatment revealed that the prevalence of retrospective reports among those with prospective observations was 48% (95% CI, 34%-62%; *I*^2^ = 96%); the prevalence of prospective observations among those who retrospectively reported childhood maltreatment was 44% (95% CI, 24%-65%; I^2^ = 99%); and the percentage of agreement between prospective and retrospective measures of childhood maltreatment was 76% (95% CI, 67%-84%; *I*^2^ = 99%). Therefore, on average, 52% of individuals with prospective observations of maltreatment did not retrospectively report it, and 56% of individuals retrospectively reporting maltreatment did not have concordant prospective observations (Figure 2A).

We next undertook sensitivity analyses to test whether the overlap between individuals identified as maltreated through prospective or retrospective measures varied as a function of the type of maltreatment (Figure 2B-E and eTable 3 in the Supplement). First, the prevalence of retrospective reports among those with prospective observations in 8 studies^13,16,17,22,23,25,27,28^ that included childhood sexual abuse was 45% (95% CI, 18%-75%; *I*^2^ = 97%); the prevalence of prospective observations among those who retrospectively reported childhood sexual abuse was 25% (95% CI, 12%-41%; *I*^2^ = 96%); and the percentage of agreement between prospective and retrospective measures of childhood sexual abuse was 86% (95% CI, 75%-94%; *I*^2^ = 99%). Second, the prevalence of retrospective reports among those with prospective observations in the 9 studies^15,16,17,23,25,26,27,28,31^ that included childhood physical abuse was 38% (95% CI, 18%-60%; *I*^2^ = 98%); the prevalence of prospective observations among those who retrospectively reported childhood physical abuse was 42% (95% CI, 19%-66%; *I*^2^ = 98%); and the percentage of agreement between prospective and retrospective measures of childhood physical abuse was 75% (95% CI, 62%-86%; *I*^2^ = 99%). Third, the prevalence of retrospective reports among those with prospective observations in the 4 studies^16,23,25,28^ that included childhood emotional abuse was 37% (95% CI, 23%-52%; *I*^2^ = 84%); the prevalence of prospective observations among those who retrospectively reported childhood emotional abuse was 15% (95% CI, 4%-33%; *I*^2^ = 97%); and the percentage of agreement between prospective and retrospective measures of childhood emotional abuse was 76% (95% CI, 57%-91%; *I*^2^ = 99%). Finally, the prevalence of retrospective reports among those with prospective observations in the 4 studies^17,19,23,25^ that included childhood neglect was 23% (95% CI, 14%-34%; *I*^2^ = 81%); the prevalence of prospective observations among those who recalled childhood neglect was 18% (95% CI, 13%-25%; *I*^2^ = 61%); and the percentage of agreement between prospective and retrospective measures of childhood neglect was 84% (95% CI, 70%-94%; *I*^2^ = 99%).

### Agreement Between Prospective and Retrospective Measures of Childhood Maltreatment

**Figure 3.**
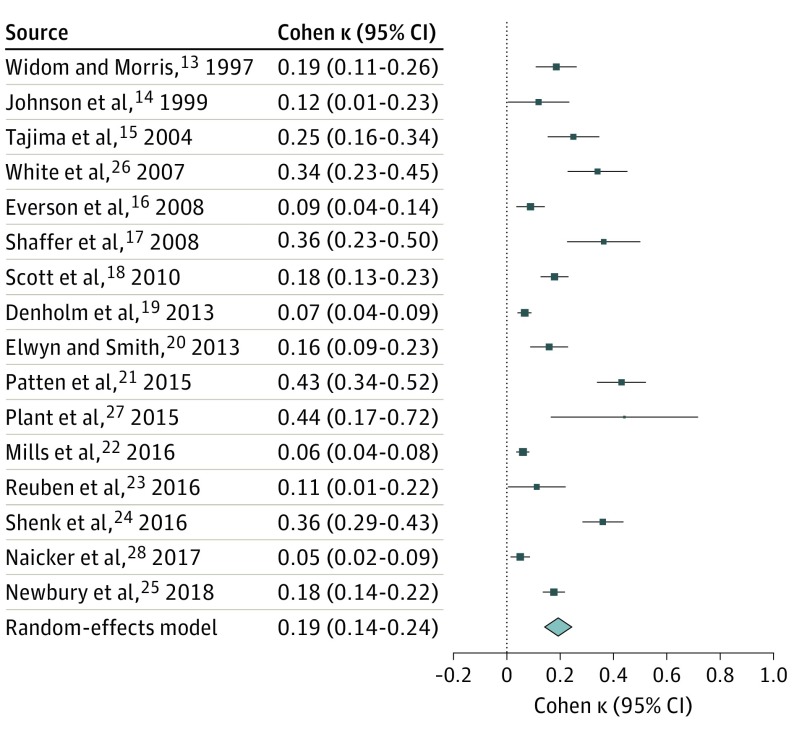
Forest Plot Depicting the Results of a Random-Effects Meta-analysis Results are reported as Cohen κ agreement between prospective and retrospective measures of childhood maltreatment. When studies reported multiple effect sizes for different maltreatment types, the mean of the κs was calculated to compute 1 overall effect size per study (κ = 0.19; 95% CI, 0.14-0.24; *P* < .001; I^2^ = 93%). Diamond marker indicates overall effect size and its variation; different sizes of markers are a function of the standard error for κs in individual studies in the random-effects model.

Because the raw percentage of agreement can be inflated by chance, we next examined the agreement between prospective and retrospective measures based on the κ, which accounts for chance findings and provides an estimate of variation in agreement in the population. A random-effects model meta-analysis of the 16 studies that included any measure of maltreatment revealed that the agreement between prospective and retrospective measures of childhood maltreatment was poor, with κ = 0.19 (95% CI, 0.14-0.24; *P* < .001; *I*^2^ = 93%). A forest plot displaying the meta-analytic findings is reported in Figure 3.

We found some evidence of publication bias, as suggested by slight asymmetry of the funnel plot (eFigure 2A in the Supplement) (Egger test, *z* = 4.4273; *P* < .001) and association between effect sizes and corresponding sampling variances (Begg test, τ = 0.37; *P* = .052). To correct for funnel-plot asymmetry arising from publication bias, we used a trim-and-fill procedure. The trim-and-fill results with 17 studies (κ = 0.19; 95% CI, 0.14-0.24; *P* < .001; *I*^2^ = 92%) (eFigure 2B in the Supplement) were similar to the results of our original meta-analysis, suggesting no substantial role of publication bias on the meta-analysis results.

Jackknife sensitivity analyses showed overall little evidence of undue effects of individual studies in the meta-analyses. The κ estimates in 16 automated permutations where each study was omitted in turn were similar and had overlapping CIs (eFigure 3 in the Supplement).

### Predictors of Heterogeneity in Agreement Between Prospective and Retrospective Measures of Childhood Maltreatment

**Figure 4.**
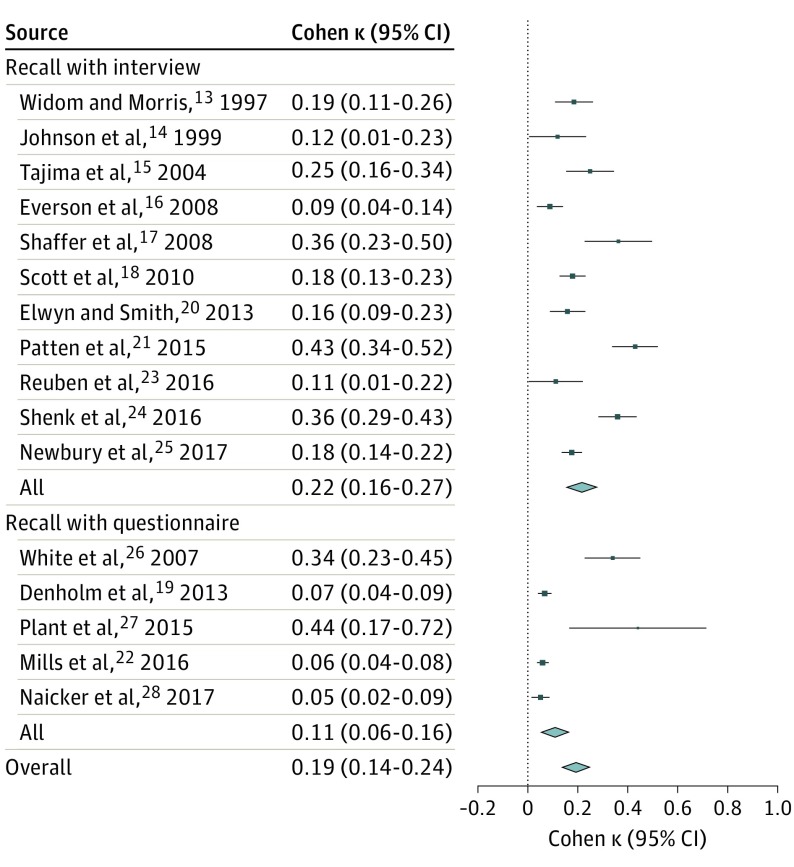
Forest Plot Depicting the Results of a Random-Effects Meta-analysis Stratified by the Type of Retrospective Measure Used Results are reported as Cohen κ agreement between prospective and retrospective measures of childhood maltreatment. Retrospective measures included interview vs questionnaire (Q = 4.1521; *df* = 1; *P* = .04). Diamond marker indicates overall effect size and its variation; different sizes of markers are a function of the standard error for κs in individual studies in the random-effects model.

Finally, we tested predictors of heterogeneity across studies with subgroup and metaregression analyses. First, we considered whether the measure used for prospective assessment of maltreatment could explain heterogeneity in effect sizes. Agreement with retrospective reports was similar regardless of whether prospective assessment was based on records (eg, child protection records or medical records; κ = 0.16; 95% CI, 0.09-0.24), reports (eg, questionnaires or interviews by parents or young people; κ = 0.22; 95% CI, 0.14-0.31), or mixed measures (records and reports; κ = 0.23; 95% CI, −0.01 to 0.48). An overall test of moderation showed that prospective measure type did not explain the heterogeneity in agreement (Q = 1.1755; *df* = 2; *P* = .56). Second, we considered whether the measure used for retrospective assessment of maltreatment could explain heterogeneity in effect sizes. As shown in Figure 4, retrospective recall during interviews (eg, verbal assessment, including reading a questionnaire aloud) showed higher agreement with prospective measures (κ = 0.22; 95% CI, 0.16-0.27) compared with retrospective recall using questionnaires (eg, written assessment; κ = 0.11; 95% CI, 0.06-0.16; difference, −0.11; *P* = .04). An overall test of moderation showed that retrospective measure type explained the heterogeneity in agreement (Q = 4.1521; *df* = 1; *P* = .04). Third, we tested whether the type of childhood maltreatment could explain heterogeneity in effect sizes. As shown in eFigure 4 in the Supplement, broad measures of childhood adversity (κ = 0.36; 95% CI, 0.25-0.48) or maltreatment (κ = 0.23; 95% CI, 0.17-0.30) showed the strongest agreement, whereas measures of emotional abuse (κ = 0.09; 95% CI, 0.04-0.13) or neglect (κ = 0.09; 95% CI, 0.05-0.13) showed the weakest agreement. A formal test of moderation across type of childhood maltreatment was not possible because the subgroups were not independent (ie, different types of childhood maltreatment were measured in the same individuals). Fourth, we tested in meta-regression analyses whether characteristics of the samples could explain heterogeneity in effect sizes. As shown in eFigure 5 in the Supplement, sample size was negatively associated with the κ coefficient (Q = 4.2251; *df* = 1; *P* = .04), indicating that smaller samples had higher agreement between prospective and retrospective measures. However, we did not find that heterogeneity in agreement was explained by other characteristics of the samples, such as sex (Q = 1.1653; *df* = 1; *P* = .28) or age at retrospective report (Q = 1.0561; *df* = 1; *P* = .30). Variation in study quality also did not explain heterogeneity in effect sizes (Q = 0.1632; *df* = 1; *P* = .69) (eTable 4 in the Supplement). Finally, in sensitivity analyses where we selected the highest effect size for the 7 studies reporting multiple effect sizes for different abuse types (instead of calculating the mean as above), we found similar results (eResults in the Supplement).

## Discussion

This meta-analysis is the first, to our knowledge, to examine the agreement between prospective and retrospective measures of childhood maltreatment. Across 16 studies that included 25 471 individuals, we found that prospective and retrospective measures of childhood maltreatment showed poor agreement. Notably, more than half of individuals with prospective observations of childhood maltreatment did not report it retrospectively, and likewise more than half of individuals retrospectively reporting childhood maltreatment did not have concordant prospective observations (Figure 2). This finding suggests that prospective and retrospective measures of childhood maltreatment identify largely different groups of individuals and, thus, cannot be used interchangeably.

Low agreement between prospective and retrospective measures of childhood maltreatment could be explained by multiple factors, such as motivation of reporters, measurement features, and memory biases. Motivation can reduce agreement if prospective or retrospective reporters may gain something by intentionally withholding information about childhood maltreatment (ie, nondisclosure, for example owing to embarrassment, feeling uncomfortable with the interviewer, not wanting to discuss upsetting events, or fear of referral to the authorities) or by fabricating information (ie, false disclosure, for example in the context of harassment, revenge, or family disputes).

Measurement features can also reduce agreement in several ways. First, all childhood maltreatment measures have imperfect test-retest reliability,^35^ and constraints on reliability add error variance, ultimately reducing agreement between prospective and retrospective measures.^36^ Second, low agreement may be due to systematic differences in the sensitivity of the measures (as reflected by the lower prevalence of childhood maltreatment identified by prospective vs retrospective measures) (eFigure 1 in the Supplement); for example, prospective measures through official records might capture only the most severe cases of maltreatment, whereas retrospective reports might detect more true cases. Third, low agreement may be owing to other systematic differences between prospective and retrospective measures, such as the reporter^13,14,15,16,17,18,19,20,21,22,23,24,25,31,32^ (eg, official records vs later self-reports), the reporting period^15,19,20,21,23^ (eg, prospective observation until 12 years of age vs retrospective recall of experiences from 0-18 years of age, or official records capturing maltreatment limited to early childhood owing to the focus of child protection services), or the definition of the maltreatment experience between prospective and retrospective measures^19^ (eg, neglect measured prospectively as lack of parental affection and retrospectively as lack of input or stimulation).

Finally, memory biases can reduce agreement by promoting underreporting and overreporting of actual experiences. On the one hand, underreporting may occur because of (1) deficits in encoding the maltreatment experience in early life owing to immature, delayed, or impaired brain development^37^; (2) deficits in consolidating the maltreatment memory owing to low emotional valence (ie, not experiencing the event as distressing)^38^ or disrupted sleep patterns^39^; (3) deficits in reconsolidating the maltreatment memory owing to memory updating during subsequent reactivation if false feedback is given^40^ (eg, being told that the experience was not abusive), if the memory is no longer associated with distressing emotions (eg, after successful psychotherapy),^41^ or if reappraisal is positively biased by personality features (eg, high agreeableness)^23^; (4) deficits in memory storage owing to brain injury or aging^42^; or (5) deficits in retrieving the maltreatment memory owing to infantile amnesia,^43^ forgetting (eg, because of low contextual reinforcement or interference by competing memories),^44^ or cognitive avoidance strategies to regulate affect.^45,46,47^ On the other hand, overreporting may occur because of (6) bias in memory encoding or reconsolidation owing to individual suggestibility (as shown in experimental paradigms of imagination inflation, false feedback, or memory implantation) or a source-monitoring error (eg, misinterpretation of internal images or dreams as lived experiences)^40,48,49^ or (7) inaccurate retrieval linked to negative bias in autobiographical memory (eg, in depression).^50^

Our findings support some of these factors. First, we found that the agreement between prospective and retrospective measures of childhood maltreatment was higher in studies that used interviews rather than questionnaires to elicit retrospective recall (Figure 4). This finding is consistent with broader observations regarding the assessment of life stress and may occur because interviews enable provision of a more detailed definition of maltreatment, contextual or anchoring methods, and greater engagement of participants.^51^ Second, agreement was also higher in studies with smaller samples (eFigure 5 in the Supplement), which might reflect the presence of more detailed retrospective assessments. Finally, the agreement for any of the childhood maltreatment measures included was substantially lower than the agreement for more clear-cut forms of adversity, such as parental loss (κ = 0.83 in the study by Reuben et al^23^; Figure 2F), suggesting that subjective interpretation of the childhood maltreatment measures may contribute to the observed heterogeneity. More research is clearly needed to disentangle factors contributing to the low agreement between prospective and retrospective measures of childhood maltreatment.

### Limitations

Our findings should be interpreted in the context of some limitations. First, because of the high levels of heterogeneity, the average meta-analytical effect sizes for agreement should be interpreted with caution. However, we used random-effects models to minimize bias linked to high heterogeneity and note that the meta-analytical CIs are narrow and consistent with the interpretation given.

Second, the results describe the agreement between prospective and retrospective measures of childhood maltreatment commonly used in the context of research studies. Therefore, the results cannot be extrapolated to infer agreement or validity of measures of childhood maltreatment used in other contexts (eg, retrospective allegations brought to the attention of the criminal justice system).

Third, although prospective measures are generally considered to be more valid (specific) indicators of the occurrence of maltreatment,^52^ the low agreement between prospective and retrospective measures cannot be interpreted to directly indicate poor validity of retrospective measures. For example, prospective measures may have lower sensitivity (ie, may identify a lower proportion of individuals who were maltreated), and the higher prevalence of retrospective measures could, thus, indicate greater ability to identify true cases of childhood maltreatment. If that was the case, predictions from retrospective measures should converge on the same outcomes as those of more specific prospective measures (convergent validity), and retrospective measures should not only be associated with outcomes assessed with the same method (ie, self-reports) but should also be associated with outcomes assessed with other methods, such as objective measures (eg, medical examinations or biomarkers [discriminant validity]).^53^ A few studies^13,15,23,25^ have tested these questions and have observed that prospective and retrospective measures assessed in the same individuals are associated with similar outcomes. However, retrospective measures showed stronger associations with self-reported outcomes than objectively assessed outcomes,^23,31^ raising concerns about potential common method bias.^54^ Therefore, further research in other samples is needed to comprehensively evaluate the construct validity of retrospective measures. Regardless of any concerns regarding their validity, retrospective reports may still be pragmatically used in the clinic as risk indicators associated with incidence of psychopathology, its course of illness, or treatment response.^3,55^

## Conclusions

Our findings have implications for researchers and health care professionals. Although retrospective reports and prospective measures identify at-risk individuals, the groups of individuals identified with either measure are not the same (Figure 2). Therefore, assuming that the underlying risk mechanisms are the same in both groups may be inaccurate. That is, the mechanisms underlying disease risk in children identified as being maltreated through prospective assessments may be different from the mechanisms underlying disease risk in adults retrospectively reporting childhood maltreatment. If risk mechanisms are different, then the 2 groups will need different interventions to effectively prevent and treat disease. As such, our findings provide a new framework for etiologic research on childhood maltreatment and intervention development.

## Notes

Supplement. **eMethods.** Systematic Review and Meta-analysis Protocol **eResults.** Study Selection and Sensitivity Analysis **eTable 1.** Checklist Summarizing Compliance With MOOSE Guidelines **eTable 2.** Checklist Summarizing Compliance With PRISMA Guidelines **eTable 3.** Summary of the Meta-analyses’ Results on the Prevalence, Overlap, and Agreement Between Prospective and Retrospective Measures Across Different Types of Childhood Maltreatment **eTable 4.** Quality Assessment of Studies With Prospective and Retrospective Measures of Childhood Maltreatment **eTable 5.** Description of Variables in Supplement 2 (Dataset of Samples With Prospective Measures of Childhood Maltreatment) **eFigure 1.** Prevalence of Childhood Maltreatment Based on 32 Paired Prospective and Retrospective Measures From 15 Studies **eFigure 2.** Funnel Plot for the Meta-analysis of the Cohen κ Agreement Between Prospective and Retrospective Measures of Childhood Maltreatment **eFigure 3.** Jackknife Sensitivity Analysis for the Meta-analysis of the Cohen κ Agreement Between Prospective and Retrospective Measures of Childhood Maltreatment **eFigure 4.** Sensitivity Analysis Showing Meta-analytic Findings on the Agreement Between Prospective and Retrospective Measures Across Different Types of Childhood Maltreatment **eFigure 5.** Meta-regression Showing Moderation of the Agreement Between Prospective and Retrospective Measures of Childhood Maltreatment by Sample Size
