# Supplementary material for: Agreement Between Prospective and Retrospective Measures of Childhood Maltreatment: A Systematic Review and Meta-analysis
Source: JAMA Psychiatry. 2019 Mar 20;76(6):584–93. doi: 10.1001/jamapsychiatry.2019.0097 (PMC6551848; doi:10.1001/jamapsychiatry.2019.0097)
Supplement: Supplement. — eMethods. Systematic Review and Meta-analysis Protocol eResults. Study Selection and Sensitivity Analysis eTable 1. Checklist Summarizing Compliance With MOOSE Guidelines eTable 2. Checklist Summarizing Compliance With PRISMA Guidelines eTable 3. Summary of the Meta-analyses’ Results on the Prevalence, Overlap, and Agreement Between Prospective and Retrospective Measures Across Different Types of Childhood Maltreatment eTable 4. Quality Assessment of Studies With Prospective and Retrospective Measures of Childhood Maltreatment eTable 5. Description of Variables in Supplement 2 (Dataset of Samples With Prospective Measures of Childhood Maltreatment) eFigure 1. Prevalence of Childhood Maltreatment Based on 32 Paired Prospective and Retrospective Measures From 15 Studies eFigure 2. Funnel Plot for the Meta-analysis of the Cohen κ Agreement Between Prospective and Retrospective Measures of Childhood Maltreatment eFigure 3. Jackknife Sensitivity Analysis for the Meta-analysis of the Cohen κ Agreement Between Prospective and Retrospective Measures of Childhood Maltreatment eFigure 4. Sensitivity Analysis Showing Meta-analytic Findings on the Agreement Between Prospective and Retrospective Measures Across Different Types of Childhood Maltreatment eFigure 5. Meta-regression Showing Moderation of the Agreement Between Prospective and Retrospective Measures of Childhood Maltreatment by Sample Size [file jamapsychiatry-76-584-s001.pdf]

Supplementary Online Content

Baldwin J, Reuben A, Newbury J, Danese A. Agreement between prospective and retrospective measures of childhood maltreatment: a systematic review and meta-analysis. *JAMA Psychiatry*. Published online March 20, 2019. doi:10.1001/jamapsychiatry.2019.0097

eMethods. Systematic Review and Meta-analysis Protocol

eResults. Study Selection and Sensitivity Analysis

eTable 1. Checklist Summarizing Compliance With MOOSE Guidelines

eTable 2. Checklist Summarizing Compliance With PRISMA Guidelines

eTable 3. Summary of the Meta-analyses' Results on the Prevalence, Overlap, and Agreement Between Prospective and Retrospective Measures Across Different Types of Childhood Maltreatment

eTable 4. Quality Assessment of Studies With Prospective and Retrospective Measures of Childhood Maltreatment

eTable 5. Description of Variables in Supplement 2 (Dataset of Samples With Prospective Measures of Childhood Maltreatment)

eFigure 1. Prevalence of Childhood Maltreatment Based on 32 Paired Prospective and Retrospective Measures From 15 Studies

eFigure 2. Funnel Plot for the Meta-analysis of the Cohen κ Agreement Between Prospective and Retrospective Measures of Childhood Maltreatment

eFigure 3. Jackknife Sensitivity Analysis for the Meta-analysis of the Cohen κ Agreement Between Prospective and Retrospective Measures of Childhood Maltreatment

eFigure 4. Sensitivity Analysis Showing Meta-analytic Findings on the Agreement Between Prospective and Retrospective Measures Across Different Types of Childhood Maltreatment

eFigure 5. Meta-regression Showing Moderation of the Agreement Between Prospective and Retrospective Measures of Childhood Maltreatment by Sample Size

This supplementary material has been provided by the authors to give readers additional information about their work.

© 2019 Baldwin J et al. *JAMA Psychiatry.*

eMethods. Systematic Review and Meta-analysis Protocol

Objective

To test the agreement between prospective and retrospective measures of childhood maltreatment.

Inclusion criteria

Study type:

All original, peer-reviewed studies (i.e., not conference abstracts) written in English and published before 1 January 2018 will be eligible to enter our meta-analysis.

Childhood maltreatment:

Definition includes maltreatment (sexual abuse, physical abuse, emotional abuse, physical neglect and emotional neglect), domestic violence, institutionalization, and bullying victimization, as well as broader measures of childhood adversity that include assessment of the above forms of maltreatment.

Prospective measure of childhood maltreatment:

Definition includes prospectively collected assessment of childhood maltreatment made whilst children were growing up (e.g., before age 18).

Retrospective measure of childhood maltreatment:

Definition includes subsequent assessment of the same individuals' exposure to childhood maltreatment made at any age.

Search methods

Data sources:

© 2019 Baldwin J et al. *JAMA Psychiatry.*

Studies will be identified initially through electronic searches in the MEDLINE, PsycINFO, Embase, and Sociological Abstracts databases. Manual search of the studies initially identified from the electronic searches and included in the analyses will be used to identify additional studies. We will identify all studies on this topic written in English and published before 1 January 2018.

Search terms/keywords:

child maltreatment, child* abuse, child* neglect, child* bull*, child* trauma, child* advers*, early life stress*

combined with:

prospective, cohort*

Data extraction

Three authors will independently extract data from eligible articles. Inconsistencies will be resolved in consensus meetings and confirmed with the authors of the primary studies when necessary. Relevant missing information will be requested from authors.

We will also collect and code information about the following variables from all studies identified with prospective assessment of childhood maltreatment:

- Sample name (if applicable)
- Sample size
- Sex distribution
- Age at latest assessment
- Childhood maltreatment definition (any maltreatment, sexual abuse, physical abuse, emotional abuse, physical neglect, emotional neglect, domestic violence, institutionalization, bullying victimization, and adverse childhood experiences)
- Prospective measure type

© 2019 Baldwin J et al. *JAMA Psychiatry.*

- Prospective measure source
- Availability of retrospective measures

If the identified studies also include retrospective measures of childhood maltreatment on the same individuals, we will then extract data on:

- Retrospective measure type
- Retrospective measure source
- Age of retrospective reporter
- Number of participants with neither prospective nor retrospective evidence of childhood maltreatment
- Number of participants with retrospective, but not prospective evidence of childhood maltreatment
- Number of participants with prospective, but not retrospective evidence of childhood maltreatment
- Number of participants with both prospective and retrospective evidence of childhood maltreatment
- Kappa indexing agreement between prospective and retrospective measures of childhood maltreatment
- Study quality will be assessed with an adapted version of the Newcastle-Ottawa Scale, which has been recommended by the Cochrane collaboration. This will include whether: the sample was representative, non-maltreated participants were drawn from the same sample as the maltreated participants, sample retention was >70% between prospective and retrospective assessments, the prospective measure was validated (e.g., based on official records or instruments that have been tested for psychometric validity and reliability), the retrospective measure was validated, the prospective and retrospective measures assessed the same time period, and the prospective and retrospective measures was based on the same source or reporter.

© 2019 Baldwin J et al. *JAMA Psychiatry.*

Data synthesis

We will use the extracted data to build contingency tables in order to compute the following outcomes:

- the prevalence of childhood maltreatment based on prospective or retrospective measures
- the conditional probability of retrospective reports among those with prospective observations
- the conditional probability of prospective observations among those with retrospective reports
- the raw percent agreement between measures
- Cohen's kappa

If the identified studies report multiple effect sizes for different childhood maltreatment types, we will average the Cohen's kappas across maltreatment types to generate one overall effect size. We will also undertake a sensitivity analysis selecting the largest kappa from each study to assess the upper limit of agreement.

We will perform random-effects meta-analyses to summarize the outcomes listed above. In the presence of significant heterogeneity in effect sizes, we will perform subgroup analyses and meta-regression analyses to test the role of selected predictors.

© 2019 Baldwin J et al. *JAMA Psychiatry.*

eResults. Study Selection and Sensitivity Analysis

Study selection

As shown in Figure 1, we identified k=7,279 articles through a search in MEDLINE, PsycINFO, Embase, and Sociological Abstracts. We reviewed the abstracts of these articles and removed those that did not assess childhood maltreatment prospectively, were literature reviews, case studies, conference proceedings, or duplicate articles (k=6,071). We then reviewed the full-texts of the remaining k=1,208 articles and excluded articles in which childhood maltreatment was not assessed prospectively or which were duplicates (k=157). We also identified additional studies from citations of identified articles (k=2). Next, we extracted data from the remaining k=1,053 articles, pooling data from k=603 articles based on overlapping samples so that each sample was represented once. This resulted in k=450 independent samples with prospective measures of childhood maltreatment (shown in eAppendix 1 [description on page 18]). Finally, we excluded samples which did not have corresponding retrospective measures of childhood maltreatment (k=428) or which did not have data on agreement between prospective and retrospective measures of childhood maltreatment that we could obtain (k=2). This led to a final total of k=20 studies with data on agreement between prospective and retrospective measures of childhood maltreatment, and k=16 with paired data (e.g., comparing prospectively identified childhood maltreatment [yes/no] with retrospectively reported childhood maltreatment [yes/no]) to allow us to compute Cohen's kappa).

Sensitivity analyses

We ran sensitivity analyses selecting the highest effect size for the studies reporting multiple effect sizes for different childhood maltreatment types (instead of averaging them).

© 2019 Baldwin J et al. *JAMA Psychiatry.*

A random-effects model meta-analysis revealed that the agreement between prospective and retrospective measures of *childhood maltreatment* was fair: kappa=0.24 (95%CI=0.18-0.31; p<0.001; I^2=95%; k=16).

We tested publication bias visually through a funnel plot and formally through funnel-plot-based tests, such as the Begg's test and the Egger's test. The effect sizes and the corresponding sampling variances were not correlated (Begg's test: tau=0.18, p=0.344) but there was some asymmetry of the funnel plot (Egger's test: z=3.2718, p=0.001) suggesting possible publication bias. To identify and correct for funnel-plot asymmetry arising from publication bias, we used a trim-and-fill procedure. The trim-and-fill results were similar to the results of our original meta-analyses (kappa=0.23, 95%CI=0.17-0.30; p<0.001; I^2=95%; k=17), suggesting no substantial role of publication bias on the meta-analysis results.

Jack-knife sensitivity analyses showed overall little evidence for undue effects of individual studies in the meta-analyses: The Cohen's kappa estimates in 16 automated permutations where each study was omitted in turn showed similar estimates and overlapping confidence intervals (kappa range=0.23-0.26).

Finally, we tested putative predictors of heterogeneity across studies with subgroup and meta-regression analyses. First, we considered if the *measure used for prospective assessment of maltreatment* could explain heterogeneity in effect sizes. Agreement with retrospective reports was similar regardless of whether prospective assessment was based on records (e.g., child protection records or medical reports; kappa=0.19, 95%CI=0.10-0.28), reports (e.g., questionnaires or interviews by parents or young people; kappa=0.27, 95%CI=0.15-0.39; difference in kappa=0.08, p=0.297), or mixed measures (both records and reports; kappa=0.39, 95%CI=0.19-0.60; difference in kappa=0.20, p=0.066). An overall test of moderation showed that prospective measure type did not explain the heterogeneity in agreement: Q(df=2)=3.6210, p=0.164. Second, we considered if the *measure used for retrospective assessment of maltreatment* could explain heterogeneity in effect sizes. Recall

© 2019 Baldwin J et al. *JAMA Psychiatry.*

during interviews showed higher agreement with prospective observations (kappa=0.27, 95%CI=0.21-0.33) compared to recall using questionnaires (kappa=0.13, 95%CI=0.07-0.20; difference in kappa=-0.14, p=0.006). An overall test of moderation showed that retrospective measure type explained the heterogeneity in agreement: Q(df=1)=7.6433, p=0.006. Third, we tested in meta-regression analyses if *characteristics of the samples* could explain heterogeneity in effect sizes. Sample size was negatively associated with Cohen's kappa (Q(df=1)= 6.2141, b=-0.00, p=0.013), indicating that smaller samples had higher agreement between prospective and retrospective measures. However, we did not find that heterogeneity in agreement was explained by other characteristics of the samples, such as proportion of female participants (Q(df=1)=0.4003, p=0.527) or age at retrospective report (Q(df=1)=0.3799, p=0.538). Finally, variation in *study quality* did not explain heterogeneity in effect sizes (Q(df=1)=0.3529, p=0.553).

© 2019 Baldwin J et al. *JAMA Psychiatry.*

eTable 1. Checklist Summarizing Compliance With MOOSE Guidelines

**Reporting background should include**	
Problem definition	X
Hypothesis statement	X
Description of study outcome(s)	X
Type of exposure or intervention used	X
Type of study designs used	X
Study population	X
**Reporting of search strategy should include**	
Qualifications of searchers (e.g. librarians and investigators)	X
Search strategy, including time period included in the synthesis and keywords	X
Effort to include all available studies, including contact with authors	X
Databases and registries searched	X
Search software used, name and version, including special features	X
Use of hand searching (e.g. reference lists of obtained articles)	X
List of citations located and those excluded including justification	x
Method of addressing articles published in languages other than English	X
Method of handling abstracts and unpublished studies	X
Description of any contact with authors	X
**Reporting methods should include**	
Description of relevance or appropriateness of studies assembled for assessing the hypothesis to be tested	X
Rationale for the selection and coding of data	X
Documentation of how data were classified and coded (eg, multiple raters, blinding, and interrater reliability)	X
Assessment of confounding	X
Assessment of study quality, including blinding of quality assessors; stratification or regression on possible predictors of study results	X
Assessment of heterogeneity	X
Description of statistical methods (eg, complete description of fixed or random effects models, justification of whether the chosen models account for predictors of study results, dose-response models, or cumulative meta-analysis) in sufficient detail to be replicated	X
Provision of appropriate tables and graphics	X
**Reporting of results should include**	
Graphic summarizing individual study estimates and overall estimate	X
Table giving descriptive information for each study included	X
Results of sensitivity testing (eg, subgroup analysis)	X
Indication of statistical uncertainty of findings	X
**Reporting of discussion should include**	
Quantitative assessment of bias (eg, publication bias)	X
Justification for exclusion (eg, exclusion of non–English-language citations)	X
Assessment of quality of included studies	X
**Reporting of conclusions should include**	
Consideration of alternative explanations for observed results	X
Generalization of the conclusions (ie, appropriate for the data presented and within the domain of the literature review)	X
Guidelines for future research	X
Disclosure of funding source	X

© 2019 Baldwin J et al. *JAMA Psychiatry.*

eTable 2. Checklist Summarizing Compliance With PRISMA Guidelines

Section/topic	#	Checklist item	Reported on page #
**TITLE**			
Title	1	Identify the report as a systematic review, meta-analysis, or both.	1
**ABSTRACT**			
Structured summary	2	Provide a structured summary including, as applicable: background; objectives; data sources; study eligibility criteria, participants, and interventions; study appraisal and synthesis methods; results; limitations; conclusions and implications of key findings; systematic review registration number.	3
**INTRODUCTION**			
Rationale	3	Describe the rationale for the review in the context of what is already known.	5
Objectives	4	Provide an explicit statement of questions being addressed with reference to participants, interventions, comparisons, outcomes, and study design (PICOS).	5
**METHODS**			
Protocol and registration	5	Indicate if a review protocol exists, if and where it can be accessed (e.g., Web address), and, if available, provide registration information including registration number.	5
Eligibility criteria	6	Specify study characteristics (e.g., PICOS, length of follow-up) and report characteristics (e.g., years considered, language, publication status) used as criteria for eligibility, giving rationale.	6
Information sources	7	Describe all information sources (e.g., databases with dates of coverage, contact with study authors to identify additional studies) in the search and date last searched.	6
Search	8	Present full electronic search strategy for at least one database, including any limits used, such that it could be repeated.	6
Study selection	9	State the process for selecting studies (i.e., screening, eligibility, included in systematic review, and, if applicable, included in the meta-analysis).	6
Data collection process	10	Describe method of data extraction from reports (e.g., piloted forms, independently, in duplicate) and any processes for obtaining and confirming data from investigators.	6-7
Data items	11	List and define all variables for which data were sought (e.g., PICOS, funding sources) and any assumptions and simplifications made.	6

© 2019 Baldwin J et al. *JAMA Psychiatry.*

Section/topic	#	Checklist item	Reported on page #
Risk of bias in individual studies	12	Describe methods used for assessing risk of bias of individual studies (including specification of whether this was done at the study or outcome level), and how this information is to be used in any data synthesis.	8
Summary measures	13	State the principal summary measures (e.g., risk ratio, difference in means).	8
Synthesis of results	14	Describe the methods of handling data and combining results of studies, if done, including measures of consistency (e.g., I^2) for each meta-analysis.	8-9
Risk of bias across studies	15	Specify any assessment of risk of bias that may affect the cumulative evidence (e.g., publication bias, selective reporting within studies).	8
Additional analyses	16	Describe methods of additional analyses (e.g., sensitivity or subgroup analyses, meta-regression), if done, indicating which were pre-specified.	9
**RESULTS**			
Study selection	17	Give numbers of studies screened, assessed for eligibility, and included in the review, with reasons for exclusions at each stage, ideally with a flow diagram.	Figure 1
Study characteristics	18	For each study, present characteristics for which data were extracted (e.g., study size, PICOS, follow-up period) and provide the citations.	22-23
Risk of bias within studies	19	Present data on risk of bias of each study and, if available, any outcome level assessment (see item 12).	eTable 4; 13
Results of individual studies	20	For all outcomes considered (benefits or harms), present, for each study: (a) simple summary data for each intervention group (b) effect estimates and confidence intervals, ideally with a forest plot.	Figure 3
Synthesis of results	21	Present results of each meta-analysis done, including confidence intervals and measures of consistency.	10-11
Risk of bias across studies	22	Present results of any assessment of risk of bias across studies (see Item 15).	11
Additional analysis	23	Give results of additional analyses, if done (e.g., sensitivity or subgroup analyses, meta-regression [see Item 16]).	12-13
**DISCUSSION**			
Summary of evidence	24	Summarize the main findings including the strength of evidence for each main outcome; consider their relevance to key groups (e.g., healthcare providers, users, and policy makers).	13, 17

© 2019 Baldwin J et al. *JAMA Psychiatry.*

Section/topic	#	Checklist item	Reported on page #
Limitations	25	Discuss limitations at study and outcome level (e.g., risk of bias), and at review-level (e.g., incomplete retrieval of identified research, reporting bias).	16-17
Conclusions	26	Provide a general interpretation of the results in the context of other evidence, and implications for future research.	16-17
**FUNDING**			
Funding	27	Describe sources of funding for the systematic review and other support (e.g., supply of data); role of funders for the systematic review.	18

From: Moher D, Liberati A, Tetzlaff J, Altman DG, The PRISMA Group (2009). Preferred Reporting Items for Systematic Reviews and Meta-Analyses: The PRISMA Statement. PLoS Med 6(7): e1000097. doi:10.1371/journal.pmed1000097

© 2019 Baldwin J et al. *JAMA Psychiatry.*

eTable 3. Summary of the Meta-analyses' Results on the Prevalence, Overlap, and Agreement Between Prospective and Retrospective Measures Across Different Types of Childhood Maltreatment

	Prevalence of prospective measures<br><br>% [95%CI]	Prevalence of retrospective measures<br><br>% [95%CI]	Prevalence of retrospective reports among those with prospective observations<br><br>% [95%CI]	Prevalence of prospective observations among those with retrospective reports<br><br>% [95%CI]	Raw percent agreement<br><br>% [95%CI]	Cohen's kappa<br><br>kappa [95%CI]
Child maltreatment	26 [11-45]	28 [14-45]	48 [34-62]	44 [24-65]	76 [67-84]	0.23 [0.17-0.30]
Child sexual abuse	7 [1-16]	10 [4-20]	45 [18-75]	25 [12-41]	86 [75-94]	0.16 [0.09-0.23]
Child physical abuse	23 [9-41]	20 [9-34]	38 [18-60]	42 [19-66]	75 [62-86]	0.17 [0.10-0.24]
Child emotional abuse	9 [1-25]	22 [8-40]	37 [23-52]	15 [4-33]	76 [57-91]	0.09 [0.04-0.13]
Child neglect	10 [4-18]	11 [3-22]	23 [14-34]	18 [13-25]	84 [70-94]	0.09 [0.05-0.13]

© 2019 Baldwin J et al. *JAMA Psychiatry.*

eTable 4. Quality Assessment of Studies With Prospective and Retrospective Measures of Childhood Maltreatment

		Sample characteristics			Validated measures		Consistency between prospective and retrospective measures		
**Author (year)**	**Study name**	**Population representative**	**Exposed and non-exposed from same population**	**Retention >70%**	**Prospective measure**	**Retrospective measure**	**Same time period assessed**	**Same source**	**Total score**
Widom (1996, 1997)[1,2]; Raphael (2001)[3]	-		✓	✓	✓	✓	✓		5
Johnson (1999)[4]	Children in the Community Study	✓	✓	✓	✓		✓		5
Tajima (2004)[5]	Lehigh Longitudinal Study		✓	✓					2
White (2007)[6]	Rutgers Health and Human Development project	✓	✓	✓			✓	✓	5
Everson (2008)[7]	Longitudinal Studies of Child Abuse and Neglect		✓	✓	✓	✓	✓		5
Shaffer (2008)[8]	Minnesota Longitudinal Study of Parents and Children		✓			✓	✓		3
Scott (2010)[9]	Te Rau Hinengaro: The New Zealand Mental Health Survey	✓	✓	✓	✓		✓		5
Denholm (2013)[10]	National Child Development Study	✓	✓						2
Elwyn (2013)[11]	Rochester Youth Development Study		✓	✓	✓				3
Patten (2015)[12]	National Longitudinal Study Survey of	✓	✓						2

© 2019 Baldwin J et al. *JAMA Psychiatry.*

Author (year)	Study name	Sample characteristics			Validated measures		Consistency between prospective and retrospective measures		Total score
		Population representative	Exposed and non-exposed from same population	Retention >70%	Prospective measure	Retrospective measure	Same time period assessed	Same source	
Plant (2015)[13]	Children and Youth/ National Population Health Survey South London Child Development Study		✓	✓	✓		✓		4
Mills (2016)[14]	Mater-University of Queensland Study of Pregnancy	✓	✓		✓		✓		4
Reuben (2016)[15]	Dunedin Multidisciplinary Health and Development Study	✓	✓	✓	✓	✓			5
Shenk (2016)[16]	Female Adolescent Development Study		✓	✓	✓	✓	✓		5
Newbury (2018)[17]	E-Risk Longitudinal Twin Study	✓	✓	✓	✓	✓	✓		6
Naicker (2017)[18]	Birth to Twenty Plus Cohort	✓	✓				✓	✓	4

Notes. Validated measures refer to those based on (i) official records (e.g., CPS; hospital/medical notes) or (ii) instruments that have been tested for psychometric validity and reliability

© 2019 Baldwin J et al. *JAMA Psychiatry.*

eFigure 1. Prevalence of Childhood Maltreatment Based on 32 Paired Prospective and Retrospective Measures From 15 Studies

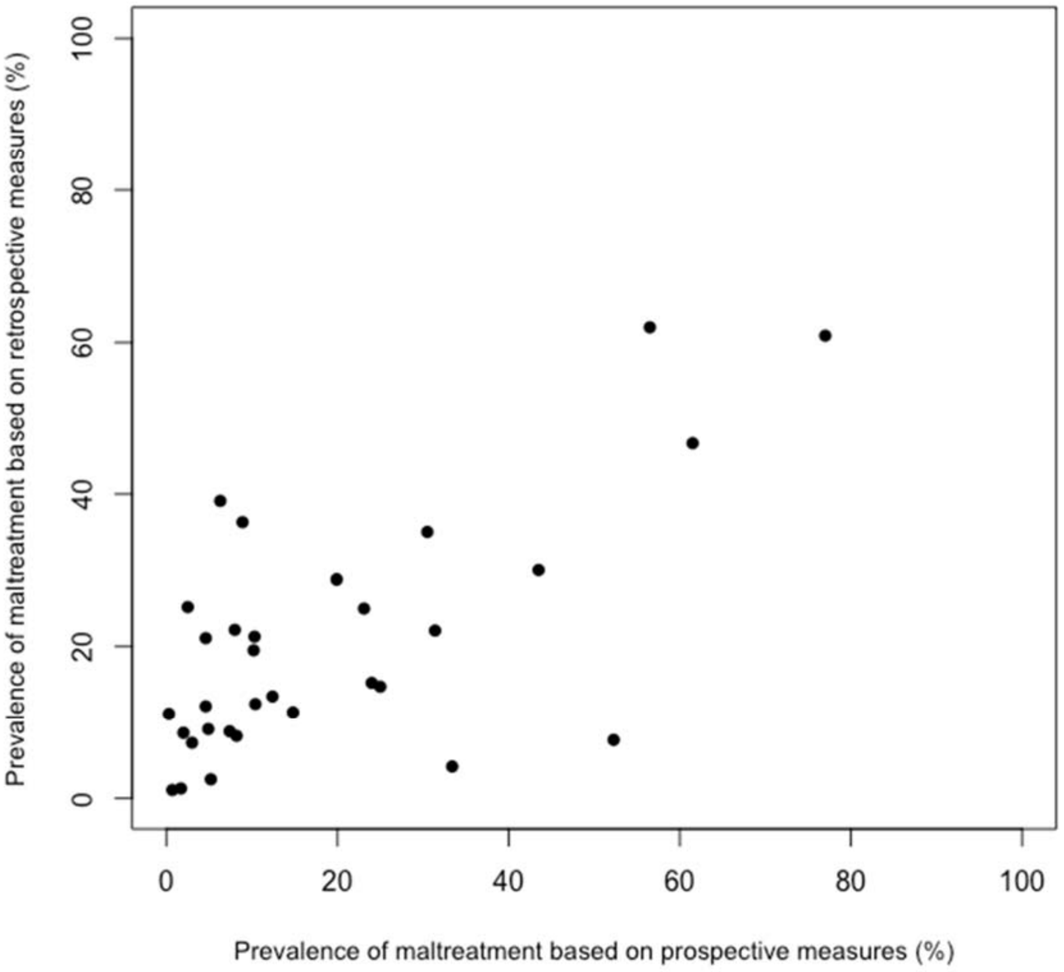

© 2019 Baldwin J et al. *JAMA Psychiatry.*

eFigure 2. Funnel Plot for the Meta-analysis of the Cohen κ Agreement Between Prospective and Retrospective Measures of Childhood Maltreatment
Panel A shows the results for the original dataset. Panel 2 shows the results after the trim and fill procedure, with one observation imputed to correct for funnel-plot asymmetry.

Panel A

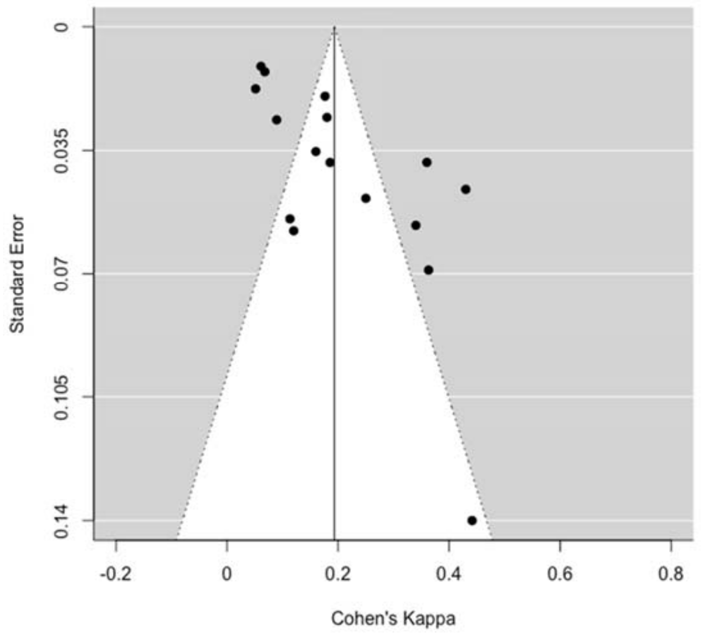

© 2019 Baldwin J et al. *JAMA Psychiatry.*

Panel B

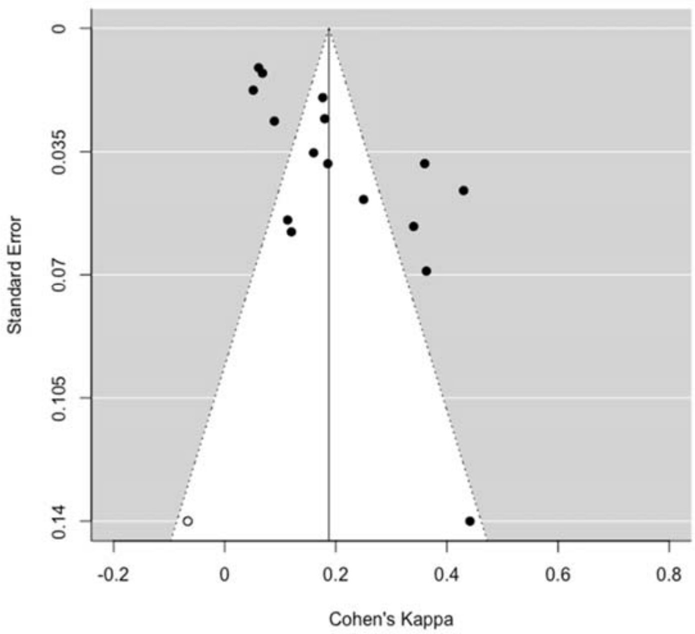

eFigure 3. Jackknife Sensitivity Analysis for the Meta-analysis of the Cohen κ Agreement Between Prospective and Retrospective Measures of Childhood Maltreatment
The estimate corresponding to each study listed reflects the Cohen's kappa value from a meta-analysis where the study was omitted.

© 2019 Baldwin J et al. *JAMA Psychiatry.*

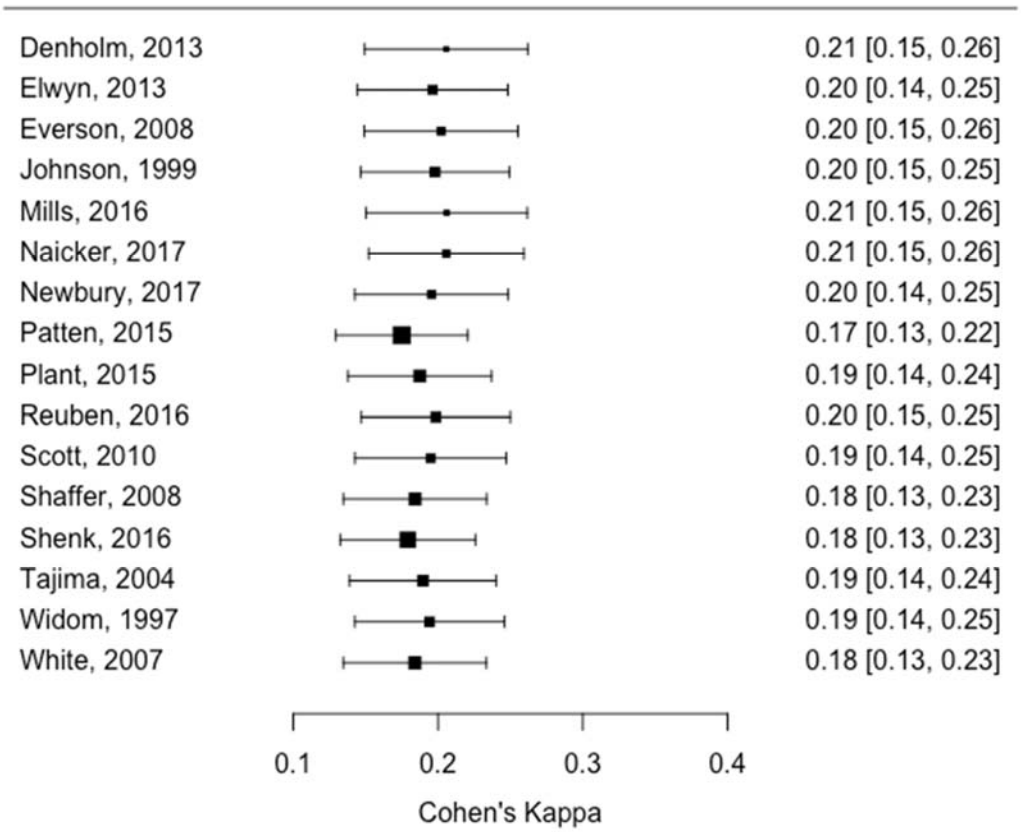

eFigure 4. Sensitivity Analysis Showing Meta-analytic Findings on the Agreement Between Prospective and Retrospective Measures Across Different Types of Childhood Maltreatment

© 2019 Baldwin J et al. *JAMA Psychiatry.*

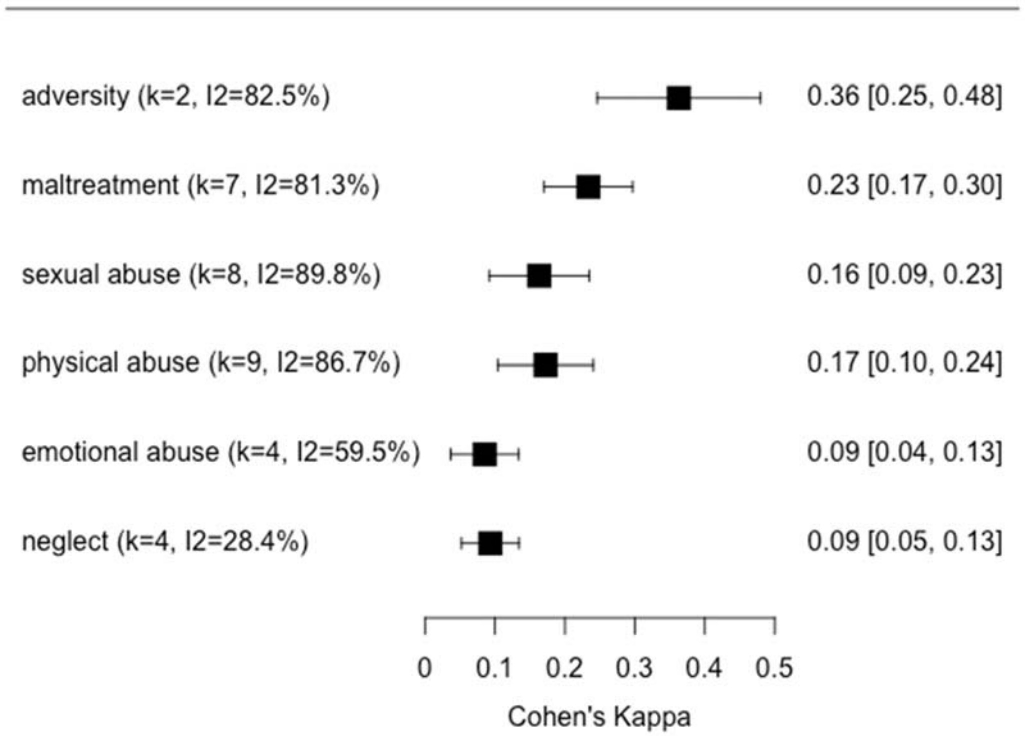

eFigure 5. Meta-regression Showing Moderation of the Agreement Between Prospective and Retrospective Measures of Childhood Maltreatment by Sample Size

© 2019 Baldwin J et al. *JAMA Psychiatry.*

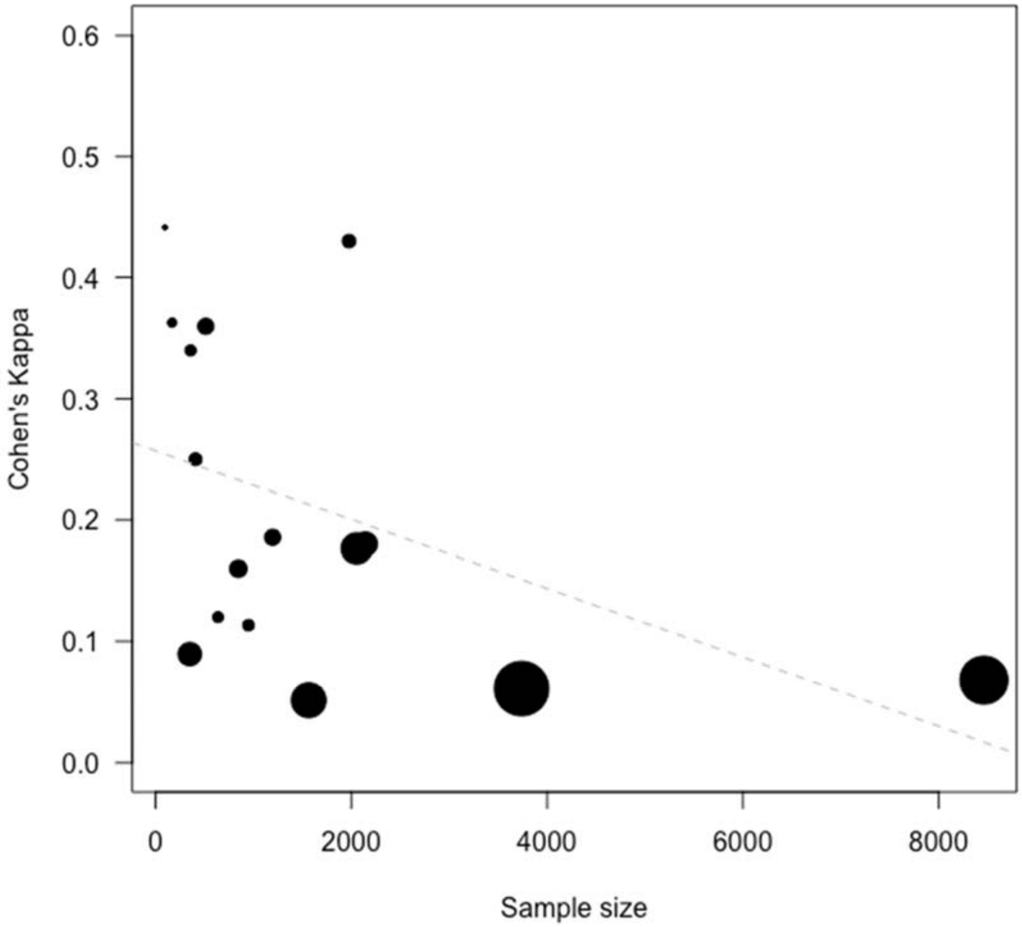

© 2019 Baldwin J et al. *JAMA Psychiatry.*

eTable 5. Description of Variables in Appendix 1 (Dataset of Samples With Prospective Measures of Childhood Maltreatment)

Variable name	Variable description	Coding
studynameknown	Whether the sample is officially named	“y” = yes<br>“n” = no
study_name	The name of the sample. If the sample does not have an official name, this is a brief description of the sample	-
example_author	The first author’s surname from an example study using that sample	-
example_year	The year of publication of the example study using that sample	-
example_link	The link to the example study using that sample	-
example_samplesize	The sample size of the example study using that sample	-
example_ageatlatestassessment	The age at latest assessment of the example study using that sample	-
example_genderfemale	The proportion of females in the example study using that sample	-
sampledescriptionnotes	A brief description of the sample	-
location	The location of the sample	-
v_type	The type of childhood adversity that was assessed	“ace” = a range of childhood adversities including maltreatment<br>“victimisation” = maltreatment + bullying<br>“maltreatment” = multiple maltreatment subtypes (e.g., physical, sexual, and emotional abuse, neglect)<br>“bullying” = bullying only<br>“physical abuse” = physical abuse only<br>“sexual abuse” = sexual abuse only<br>“emotional abuse” = emotional abuse only<br>“neglect” = physical or emotional neglect only<br>“domestic violence” = domestic violence only<br>“institutionalisation” = institutionalisation only

© 2019 Baldwin J et al. *JAMA Psychiatry.*

Description of variables in Appendix 1 (cont.)

Variable name	Variable description	Coding
v_assessment	The method of prospective assessment of childhood maltreatment	"welfare" = Child Protection Services records<br>"hospital" = hospital records<br>"interview" = interview<br>"questionnaire" = questionnaire<br>"mixed" = welfare/hospital records & interview/questionnaire<br>"multiple" = interview & questionnaire
v_reporter	The reporter/source of the prospective assessment of childhood maltreatment	"mixed" = welfare/hospital records & another reporter (e.g., parent, child, teacher)<br>"multiple" = multiple reporters (e.g., parent, child, teacher)<br>"records" = welfare/hospital records<br>"parent" = parent<br>"self" = child<br>"teacher" = teacher
retro_data	Whether corresponding retrospective measures of childhood maltreatment are available	"y" = yes<br>"n" = no
link_pro_retro_study	Link to a study including both prospective and retrospective measures of childhood maltreatment	-

Note. Many different studies using prospective measures of childhood maltreatment were based on the same sample, and we pooled information across studies so that each sample was represented once in the dataset. However, for each sample we provide an example study. These example studies may not reflect the full breadth of all of the prospective measures of childhood maltreatment available for that sample.

© 2019 Baldwin J et al. *JAMA Psychiatry.*

References:

1. Widom CS, Morris S. Accuracy of adult recollections of childhood victimization, part 2: childhood sexual abuse. *Psychol Assess.* 1997;9(1):34.
2. Widom CS, Shepard RL. Accuracy of adult recollections of childhood victimization: Part 1. Childhood physical abuse. *Psychol Assess.* 1996;8(4):412.
3. Raphael KG, Widom CS, Lange G. Childhood victimization and pain in adulthood: a prospective investigation. *Pain.* 2001;92(1-2):283-293.
4. Johnson JG, Cohen P, Brown J, Smailes EM, Bernstein DP. Childhood maltreatment increases risk for personality disorders during early adulthood. *Arch Gen Psychiatry.* 1999;56(7):600-606.
5. Tajima EA, Herrenkohl TI, Huang B, Whitney SD. Measuring child maltreatment: A comparison of prospective parent reports and retrospective adolescent reports. *Am J Orthopsychiatry.* 2004;74(4):424.
6. White HR, Widom CS, Chen P-H. Congruence between adolescents' self-reports and their adult retrospective reports regarding parental discipline practices during their adolescence. *Psychol Rep.* 2007;101(3_suppl):1079-1094.
7. Everson MD, Smith JB, Hussey JM, et al. Concordance between adolescent reports of childhood abuse and child protective service determinations in an at-risk sample of young adolescents. *Child Maltreat.* 2008;13(1):14-26.
8. Shaffer A, Huston L, Egeland B. Identification of child maltreatment using prospective and self-report methodologies: A comparison of maltreatment incidence and relation to later psychopathology. *Child Abuse Neglect.* 2008;32(7):682-692.
9. Scott KM, Smith DR, Ellis PM. Prospectively ascertained child maltreatment and its association with DSM-IV mental disorders in young adults. *Arch Gen Psychiatry.* 2010;67(7):712-719.
10. Denholm R, Power C, Li L. Adverse childhood experiences and child-to-adult height trajectories in the 1958 British birth cohort. *Int J Epidemiol.* 2013;42(5):1399-1409.
11. Elwyn L, Smith C. Child maltreatment and adult substance abuse: The role of memory. *Journal of Social Work Practice in the Addictions.* 2013;13(3):269-294.
12. Patten S, Wilkes T, Williams J, et al. Retrospective and prospectively assessed childhood adversity in association with major depression, alcohol consumption and painful conditions. *Epidemiology and Psychiatric Sciences.* 2015;24(2):158-165.
13. Plant DT, Pariante CM, Sharp D, Pawlby S. Maternal depression during pregnancy and offspring depression in adulthood: role of child maltreatment. *The British Journal of Psychiatry.* 2015;207(3):213-220.
14. Mills R, Kisely S, Alati R, Strathearn L, Najman J. Self-reported and agency-notified child sexual abuse in a population-based birth cohort. *J Psychiatr Res.* 2016;74:87-93.
15. Reuben A, Moffitt TE, Caspi A, et al. Lest we forget: comparing retrospective and prospective assessments of adverse childhood experiences in the prediction of adult health. *J Child Psychol Psychiatry.* 2016;57(10):1103-1112.
16. Shenk CE, Noll JG, Peugh JL, Griffin AM, Bensman HE. Contamination in the prospective study of child maltreatment and female adolescent health. *J Pediatr Psychol.* 2015;41(1):37-45.
17. Newbury JB, Arseneault L, Moffitt TE, et al. Measuring childhood maltreatment to predict early-adult psychopathology: comparison of prospective informant-reports and retrospective self-reports. *J Psychiatr Res.* 2018;96:57-64.
18. Naicker SN, Norris SA, Mabaso M, Richter LM. An analysis of retrospective and repeat prospective reports of adverse childhood experiences from the South African Birth to Twenty Plus cohort. *PLoS One.* 2017;12(7):e0181522.

© 2019 Baldwin J et al. *JAMA Psychiatry.*
